# Short-Term Dapagliflozin Administration in Autosomal Dominant Polycystic Kidney Disease—A Retrospective Single-Arm Case Series Study

**DOI:** 10.3390/jcm12196341

**Published:** 2023-10-03

**Authors:** Fumiyuki Morioka, Shinya Nakatani, Hideki Uedono, Akihiro Tsuda, Katsuhito Mori, Masanori Emoto

**Affiliations:** 1 Department of Metabolism, Endocrinology and Molecular Medicine, Graduate School of Medicine, Osaka City University, Osaka 545-8585, Japan; j21574d@omu.ac.jp (F.M.); uedono1217@yahoo.co.jp (H.U.); naranotsudadesu@infoseek.jp (A.T.); emoto-m@omu.ac.jp (M.E.); 2 Department of Nephrology, Osaka Metropolitan University Graduate School of Medicine, Osaka 545-8585, Japan; ktmori@omu.ac.jp

**Keywords:** autosomal dominant polycystic kidney disease, dapagliflozin, total kidney volume

## Abstract

Treatment with sodium-glucose cotransporter-2 (SGLT2) inhibitors may have pleiotropic and beneficial effects in terms of ameliorating of risk factors for the progression of autosomal dominant polycystic kidney disease (ADPKD). However, there is insufficient evidence regarding the use of these drugs in patients with ADPKD, as they were excluded from several clinical trials conducted to explore kidney protection provided by SGLT2 inhibitors. This retrospective single-arm case series study was performed to investigate the effects of dapagliflozin, a selective SGLT2 inhibitor administered at 10 mg/day, on changes in height-adjusted kidney volume (htTKV) and estimated glomerular filtration rate (eGFR) in ADPKD patients. During a period of 102 ± 20 days (range 70–156 days), eGFR was decreased from 47.9 (39.7–56.9) to 40.8 (33.7–44.5) mL/min/1.73 m^2^ (*p* < 0.001), while htTKV was increased from 599 (423–707) to 617 (446–827) mL/m (*p* = 0.002) (n = 20). The annual increase in htTKV rate was significantly promoted, and urinary phosphate change was found to be correlated with the change in htTKV (rs = 0.575, *p* = 0.020). In the examined patients, eGFR was decreased and htTKV increased during short-term administration of dapagliflozin. To confirm the possibility of the effects of dapagliflozin on ADPKD, additional interventional studies are required.

## 1. Introduction

Autosomal dominant polycystic kidney disease (ADPKD) is characterized by progressive development and enlargement of kidney cysts, the fourth greatest cause worldwide of kidney failure in patients who have undergone kidney replacement therapy [1,2]. Furthermore, several previous clinical studies have revealed that fasting plasma glucose levels [3], obesity [4], and hypertension [5,6] are risk factors for kidney enlargement and function deterioration in ADPKD patients. In vivo results have also demonstrated that a ketogenic diet and oral administration of the ketone *β*-hydroxybutyrate inhibit kidney enlargement [7].

Sodium-glucose cotransporter-2 (SGLT2) inhibitors were originally introduced as a novel class of antidiabetic drugs, while several recent clinical trials have shown that they have heart and kidney protective effects in chronic kidney disease (CKD) patients both with and without diabetes [8,9,10]. In the Dapagliflozin in Patients with Chronic Kidney Disease (DAPA-CKD) trial, dapagliflozin was administered to CKD patients with an estimated glomerular filtration rate (eGFR) of 25 to 75 mL/min/1.73 m^2^ and a urinary albumin-to-creatinine ratio of 200 to 5000 mg/gCr [8]. Additionally, Empagliflozin in the Patients with Chronic Kidney Disease (EMPA-Kidney) trial, empagliflozin was given to those with an eGFR of 20 to 45 mL/min/1.73 m^2^, or an eGFR of at least 45 to 90 mL mL/min/1.73 m^2^, and a urinary albumin-to-creatinine ratio of at least 200 mg/gCr [10]. Results of the DAPA-CKD trial showed a greater reduction in eGFR in the dapagliflozin group as compared to the placebo group (−3.97 ± 0.15 vs. −0.82 ± 0.15 mL/min/1.73 m^2^) during the first two weeks, namely an initial dip [8], while an initial dip was also recognized in the EMPA-Kidney trial [10]. Thus, it is considered that a favorable and reversible hemodynamic change in the glomerulus is possible with the use of an SGLT2 inhibitor. In the DAPA-CKD trial, a smaller annual change in mean eGFR was observed in the dapagliflozin group as compared with the placebo group (−1.67 ± 0.11 and −3.59 ± 0.11 mL/min/1.73 m^2^, respectively) at two weeks after initiation of treatment. Additionally, the eGFR slope from baseline to 30 months in the dapagliflozin and placebo groups was −2.86 ± 0.11 and −3.79 ± 0.11 mL/min/1.73 m^2^ per year, respectively [8]. Results indicating a decline in eGFR from two months to the final follow-up examination in the EMPA-Kidney trial showed a between-group difference of 1.37 mL/min/1.73 m^2^ (95% CI, 1.16 to 1.59) per year [10]. In a pre-specified analysis of DAPA-CKD trial subjects, effects of dapagliflozin on eGFR were observed not only in participants with type 2 diabetes but also in those affected by glomerulonephritis, including IgA nephropathy [11]. Thus, SGLT2 inhibitors are now widely prescribed to CKD patients with various conditions to slow kidney function decline. Additionally, SGLT2 inhibitors have been reported to reduce body weight and blood pressure, risk factors for the progression of ADPKD, in non-diabetic patients with CKD [10] and also in those with heart failure [12], while in vivo results have shown increased endogenous ketone body levels [13]. Thus, it is considered possible that SGLT2 inhibitors have benefits for patients with ADPKD in terms of kidney protection. 

Cardiovascular disease (CVD) is the major cause of mortality in ADPKD cases and contributes to a significant disease burden [14,15,16,17]. Patients with ADPKD experience CVD events with greater severity and have an increased risk of CVD-related death as compared to the general population, with 33% of those mortalities mainly due to ischemic heart disease or congestive heart failure [18]. Furthermore, results from the DAPA-CKD and EMPA-Kidney trials demonstrated the clinical benefits of SGLT2 inhibitors for improving CVD outcomes in CKD patients [8,10]. Thus, SGLT2 inhibitors may have benefits for patients with ADPKD in terms of preventing CVD events.

Tolvaptan, a vasopressin V2 receptor (V2R) antagonist, is recognized as a disease-specific treatment option for ADPKD [19]. It is also known that its administration can lead to a rapid decline in eGFR induced by hemodynamic change in the glomerulus [20]. Thus, cotreatment with an SGLT2 inhibitor, and tolvaptan may have increased diuretic effects and cause glomerular hemodynamic changes and subsequent acute kidney injury (AKI). Previously presented metanalysis findings [21] and also those of a pre-specified analysis of DAPA-CKD trial subjects [22] revealed that SGLT 2 inhibitors, including dapagliflozin, reduced the risk of an abrupt decline in kidney function in CKD patients. However, the interactions between SGLT2 inhibitors and tolvaptan have not been well investigated. 

Several studies have used murine models of polycystic kidney disease (PKD) to evaluate the effects of SGLT2 inhibition. Five weeks of treatment with phlorizin, which has inhibitory effects on both sodium-glucose cotransporter-1 (SGLT1) and SLGT2, inhibited cyst growth in Han:SPRD rats [23]. Furthermore, dapagliflozin, a selective SGLT2 inhibitor, improved kidney function and albuminuria in Han:SPRD rats, though it failed to slow cyst growth [24]. Surprisingly, another study found that dapagliflozin administration led to increased cyst volume in PCK rats [25]. Thus, results from the studies of SGLT2 inhibitors using animal models of PKD have provided conflicting results.

To date, there is insufficient evidence regarding the use of these drugs in patients with ADPKD, as those were excluded from several clinical trials conducted to explore the kidney protection provided by SGLT2 inhibitors [8,10]. Currently, dapagliflozin is approved for patients with CKD in Japan. However, whether it is beneficial or harmful for ADPKD patients in terms of kidney function preservation is unclear. Hence, the present short-term retrospective observational study of patients with ADPKD was conducted to investigate the effects of dapagliflozin on changes in eGFR and height-adjusted kidney volume (htTKV).

## 2. Materials and Methods

### 2.1. Ethics Statement

This study was performed according to ethical guidelines for medical and health research involving human subjects provided by the Japanese Ministry of Health, Labor, and Welfare, as well as the Declaration of Helsinki. The protocol of this investigation was approved by the Ethics Committee of Osaka Metropolitan University (No. 2022-081).

### 2.2. Patients

**Figure 1:**
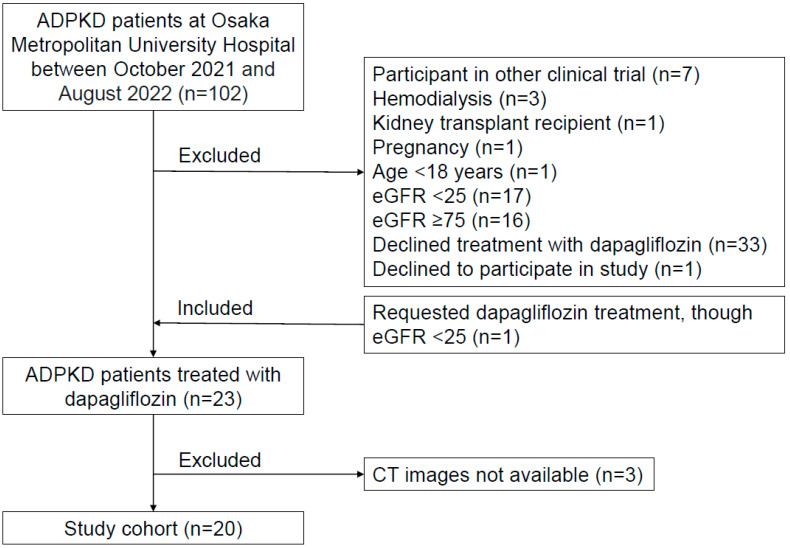
Flow chart of the study. A total of 20 patients who received dapagliflozin treatment and had CT images available were enrolled.

A flow chart of the study participants is presented in Figure 1. A total of 102 ADPKD patients who were regularly examined by nephrologists at Osaka Metropolitan University Hospital between October 2021 and August 2022 were initially considered subjects. ADPKD was diagnosed using previously described criteria [26]. Those undergoing hemodialysis (n = 3), with prior kidney transplantation (n = 1), pregnant (n = 1), under 18 years of age (n = 1), or who were participants in other clinical trials (n = 7) were excluded. Furthermore, ADPKD patients with an eGFR <25 or >75 mL/min/1.73 m^2^ were generally excluded, according to criteria noted in a previous study [10], though one whose eGFR was 24.6 mL/min/1.73 m^2^ strongly requested dapagliflozin treatment, thus being included. Thirty-three of the remaining 56 patients declined to receive dapagliflozin treatment after an explanation regarding a lack of specific data showing effects for ADPKD and also potential side effects, including ketoacidosis, volume depletion, urosepsis, and pyelonephritis. Twenty-three patients were selected to receive dapagliflozin, administered at 10 mg/day. Of those, 20 who underwent computed tomography (CT) scanning between October 2021 and August 2022, at the start of dapagliflozin administration, were enrolled. 

### 2.3. Physical and Laboratory Measurements and Other Clinical Information

All blood and urine samples were collected in the morning after overnight fasting on the same day as the CT examinations. Laboratory measurements were performed using routine assays with an automated method [27]. Hemoglobin A1c was assessed using the National Glycohemoglobin Standardization Program (NGSP) equivalent value according to the guidelines of the Japan Diabetes Society [28]. The diagnosis of type 2 diabetes mellitus was based on medical records and criteria for diabetes mellitus defined in the Report of the Expert Committee on the Diagnosis and Classification of Diabetes Mellitus [29]. Kidney function was assessed by eGFR using a formula for Japanese individuals [30]. When serum albumin was <4.0 g/dL, then the following formula was used: corrected calcium (mg/dL) = measured Ca (mg/dL) + 4 − measured albumin (g/dL) [31]. Urinary protein and phosphate levels were normalized to those of creatinine (Cr), and then expressed as g/gCr, as previously described [32]. 

Age, gender, height, weight, family history, complications of ADPKD (liver cysts, intracranial aneurysms), and tolvaptan administration details were collected by a review of their medical records.

### 2.4. Computed Tomography Imaging for Total Kidney Volume (TKV)

CT imaging was used for kidney examinations in the enrolled patients. Total kidney volume (TKV) was estimated based on linear dimensions using an ellipsoid formula, as follows: length × width × thickness × π/6 [33,34,35]. Annual changes were calculated as shown below. At our department, an abdominal CT examination is generally performed for ADPKD patients every two to three years. Furthermore, that is also performed for such patients within two to four months after starting the administration of a new drug to determine its efficacy and safety.

#### 2.4.1. Pre-Dapagliflozin Treatment

Annual change in TKV rate (%/year) = [(TKV at most recent examination before starting dapagliflozin) − (TKV at second most recent examination before starting dapagliflozin)]/[(TKV at second recent before starting dapagliflozin)/(days between CT examinations/365 days)] × 100.

#### 2.4.2. Post-Dapagliflozin Treatment

Annual change in TKV rate (%/year) = [(TKV after dapagliflozin administration) − (TKV at start of administration of dapagliflozin)]/[(TKV at start of dapagliflozin administration)/(days between CT examinations/365 days)] × 100.

### 2.5. Statistical Analysis

Continuous variables are expressed as medians (interquartile ranges) and categorical variables as numbers (percentages). A paired Student’s *t*-test or Wilcoxon signed-rank test was used for comparisons of clinical parameters of ADPKD patients who received dapagliflozin between pre-administration and follow-up examinations. A correlation analysis was conducted to examine the relationships between annual changes in htTKV rate and changes in clinical parameters during dapagliflozin treatment. Statistical analyses were performed using the JMP software package, version 10 (SAS Institute, Inc., Cary, NC, USA). *p* values < 0.05 were considered to indicate statistical significance.

## 3. Results

### 3.1. Clinical Characteristics of ADPKD Patients

The baseline clinical characteristics of the present ADPKD patients are shown in Table 1. Nine (45%) were male, and the median age at the initiation of dapagliflozin was 51 (46–57) years. Only two (10%) patients did not have a family history of ADPKD. Body mass index was 23.0 (20.9–24.8) kg/m^2^, with one (5%) affected by diabetes. eGFR, serum phosphate, and calcium levels were 47.9 (39.7–56.9) mL/min/1.73 m^2^, 3.5 (3.3–3.7) mg/dL, and 9.4 (9.2–9.6) mg/dL, respectively. The numbers of patients receiving tolvaptan, renin-angiotensin-aldosterone system inhibitor, calcium channel blocker, beta blocker, anti-diabetic agent, or phosphorus binder treatments at the time of the baseline examination were 11 (55%), 11 (55%), 12 (60%), 4 (20%), 1 (5%), and 1 (5%), respectively. During the dapagliflozin administration period, none made a change in regard to those other medications. 

### 3.2. Changes in Clinical Parameters after Dapagliflozin Treatment

**Figure 2:**
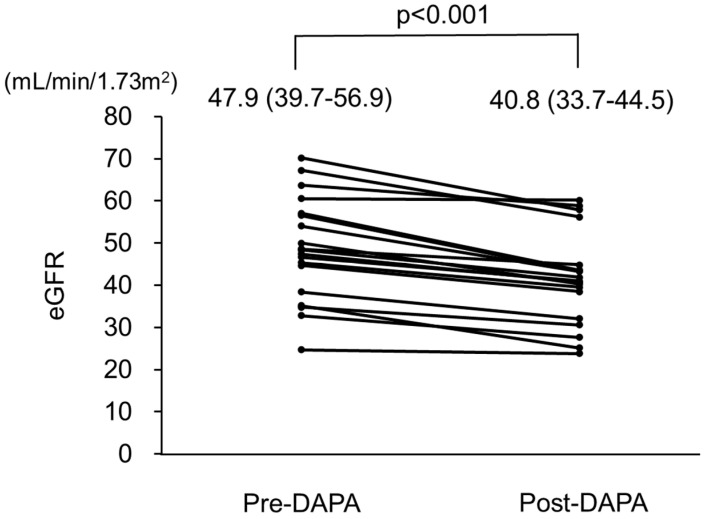
Changes in estimated glomerular filtration rate (eGFR) in patients with autosomal dominant polycystic kidney disease (ADPKD) treated with dapagliflozin. eGFR was significantly decreased after treatment with dapagliflozin. Pre-DAPA: pre-dapagliflozin treatment; Post-DAPA: post-dapagliflozin treatment.

Changes in clinical parameters after administration of dapagliflozin are presented in Table 2. During a total period of 102 ± 20 days (range 70–156 days), eGFR decreased from 47.9 (39.7–56.9) mL/min/1.73 m^2^ at the baseline to 40.8 (33.7–44.5) mL/min/1.73 m^2^ at the end of the observation period (*p* < 0.001). Changes in eGFR in all patients are shown in Figure 2 and Table 3. Furthermore, hemoglobin and serum creatinine levels were significantly increased [13.1 (12.2–14.4) to 14.0 (13.1–15.1) g/L (*p* <0.001) and 1.13 (0.93–1.34) to 1.37 (1.05–1.55) mg/dL (*p* < 0.001)], respectively (Table 2). Blood pressure, serum albumin, calcium, phosphate, urinary protein, and urinary phosphate levels were not significantly different between the baseline and final follow-up examinations.

### 3.3. Changes in eGFR before and after Dapagliflozin Treatment

**Figure 3:**
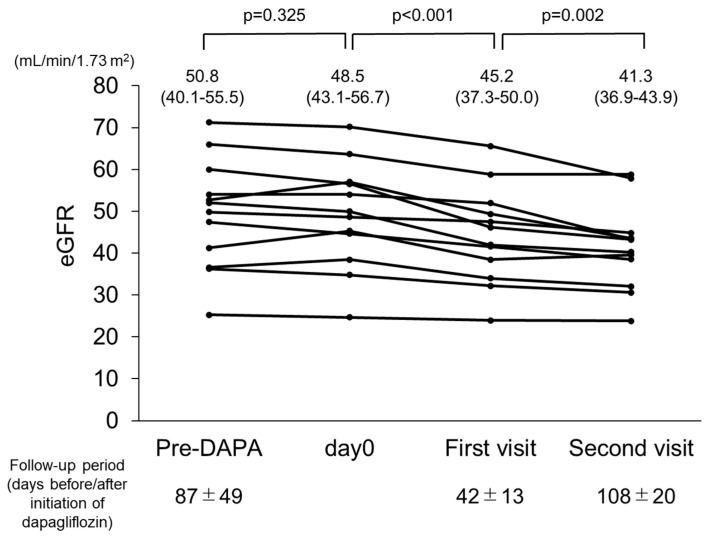
Changes in estimated glomerular filtration rate (eGFR) in patients with autosomal dominant polycystic kidney disease (ADPKD) treated with dapagliflozin (n = 14). eGFR was significantly decreased during treatment with dapagliflozin.

In consideration of the initial dip in eGFR after dapagliflozin administration, values obtained at the most recent evaluation before starting dapagliflozin, on the day of starting dapagliflozin administration, and one month after starting administration were examined (n = 14) (Figure 3). From the first day of dapagliflozin administration until the first visit one month later, eGFR was reduced from 48.5 to 45.2 mL/min/1.73 m^2^, which can be considered the initial dip. At the second visit after starting dapagliflozin administration, a significant decline was noted (*p* = 0.002). In contrast, no such significant decline was seen during the pre-dapagliflozin treatment period from the final evaluation before starting dapagliflozin until the first day of administration (*p* = 0.325).

### 3.4. Changes in htTKV before and after Dapagliflozin Treatment

**Figure 4:**
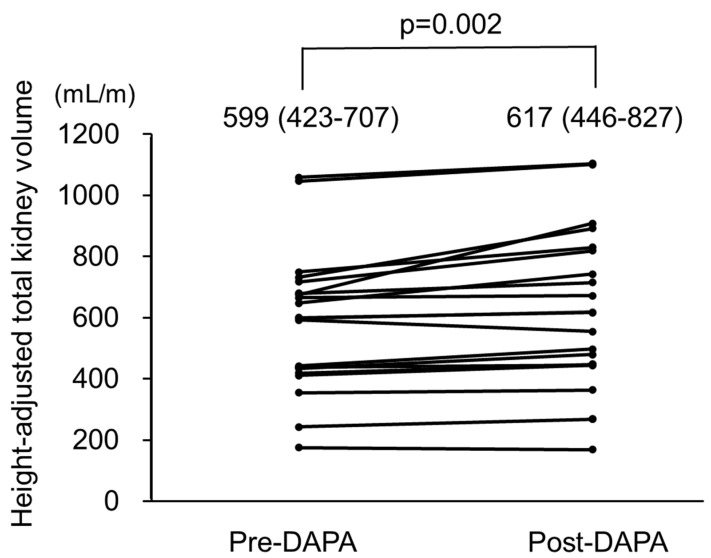
Height-adjusted total kidney volume (htTKV) in autosomal dominant polycystic kidney disease (ADPKD) patients treated with dapagliflozin. htTKV was significantly increased after treatment with dapagliflozin. pre-DAPA: pre-dapagliflozin treatment; post-DAPA: post-dapagliflozin treatment.

Changes in htTKV in all patients are shown in Figure 4 and Table 3. During the observational period, htTKV significantly increased from 599 (423–707) to 617 (446–827) mL/m (*p* = 0.002), with findings of a representative case presented in Supplemental Figure S1.

### 3.5. Changes in eGFR and htTKV in ADPKD Patients with and without Tolvaptan Treatment

In ADPKD patients without tolvaptan treatment (n = 9), eGFR was decreased from 46.7 (44.2–60.6) mL/min/1.73 m^2^ at the baseline to 40.7 (39.0–49.7) mL/min/1.73 m^2^ at the end of the observation period (*p* = 0.004), while htTKV was significantly increased from 598 (387–678) to 619 (404–767) mL/m (*p* = 0.008). In those patients with tolvaptan treatment (n = 11), eGFR was decreased from 48.6 (34.8–57.0) mL/min/1.73 m^2^ at the baseline to 41.9 (30.6–44.8) mL/min/1.73 m^2^ at the end of the observation period (*p* = 0.001), and htTKV was significantly increased from 600 (434–750) to 616 (479–892) mL/m (*p* = 0.007).

### 3.6. Changes in Annual htTKV Rate before and after Dapagliflozin Treatment

The annual change in htTKV rate before dapagliflozin treatment was −1.4% (−11.9–5.0%) during a total period of 725 ± 569 days (range 91–1750 days), while that after the start of dapagliflozin treatment was 23.9% (10.2–44.7%) (Supplemental Figure S2). During the treatment period, the annual change in htTKV rate was significantly increased (*p* < 0.001).

### 3.7. Correlations between Changes in htTKV and Changes in Clinical Parameters during Dapagliflozin Treatment 

Correlations between changes in htTKV and changes in various clinical parameters are shown in Table 4. While eGFR and serum phosphate levels were not significantly correlated with changes in htTKV, urinary phosphate level changes showed a significant positive correlation (r_s_ = 0.575, *p* = 0.020) (Table 4, Supplemental Figure S3).

## 4. Discussion

The present study was conducted to investigate whether dapagliflozin is beneficial or harmful for patients with ADPKD in regard to changes in eGFR and htTKV. In the enrolled participants, short-term administration of dapagliflozin was associated with decreased eGFR and increased htTKV. Considering our findings, prudent informed consent, including written consent, is necessary when administering dapagliflozin to ADPKD patients. 

At two to four weeks after SGLT2 inhibitor administration, a modest dip in eGFR was observed in CKD patients with diabetes, and an acute dip was seen in those without diabetes [36]. In the DAPA-CKD trial, CKD patients, whose eGFR was 43.2 mL/min/1.73 m^2^, showed a dip in that ratio of approximately 10% [8]. In the present study, at one month after administration of dapagliflozin, eGFR was reduced from 48.5 to 45.2 mL/min/1.73 m^2^ in 14 of the ADPKD patients, similar to the reduction seen in that trial, which can be explained as an initial dip caused by dapagliflozin. In the DAPA-CKD trial, after the initial dip, eGFR decline became mild in those CKD patients, whereas eGFR continued to show a decrease in the present ADPKD patients up to approximately three months after administration of dapagliflozin. This discrepancy in rate of eGFR decline after the initial dip between that trial and the present study may have been due to increased cyst volume after dapagliflozin administration. 

Impairment of glucose metabolism has been highlighted as a key feature and important disease modulator in ADPKD cases [37] and reported to be associated with cystogenesis in vitro [38,39] and in vivo [40,41]. Under normoglycemia, up to 97% of filtered glucose is reabsorbed via SGLT2 in early proximal tubules, while the remaining less than 3% is reabsorbed by SGLT1 in late proximal tubules [42]. In instances of SGLT2 knockout or inhibition, approximately 40% of glucose has been reported to be reabsorbed by SGLT1 [43,44]. It has also been reported that dual inhibition of SGLT1 and SGLT2 by phlorizin decreased cyst growth in Han:SPRD rats [23], whereas dapagliflozin was not associated with a decrease in cyst growth in those rats [24], suggesting that increased glucose reabsorption via SGLT1 by treatment with an SGLT2 inhibitor possibly increases cyst growth in late proximal tubules. 

It has been speculated that increased intratubular osmotic pressure caused by increased glucose concentration in late proximal tubules was a factor that promoted cyst enlargement in PCK rats treated with dapagliflozin [25]. In addition, a recent study that used a human organoid-on-chip model of PKD demonstrated that fluid flow and solute concentrations can be positive regulators of cyst expansion [38]. In contrast, tolvaptan, a vasopressin V_2_-receptor antagonist used for disease-specific treatment worldwide, was found to significantly reduce urinary osmolality [20], while another study showed that a greater decrease in urinary osmolality in subjects treated with tolvaptan was associated with slower eGFR decline [45]. These findings indicated that urinary osmotic pressure increased by the use of SGLT2 inhibitors can promote cystogenesis in late proximal tubules. 

Recently, findings presented by the Consortium for Radiologic Imaging Studies of PKD (CRISP) regarding fibroblast growth factor 23 (FGF23), a phosphaturic hormone [46], demonstrated its association with the rate of increase in htTKV and that of decline in GFR in APDKD patients [47]. Although the mechanism of FGF23 for acceleration of ADPKD progression was unclear in that study, we speculate that calcium-phosphate crystal depositions mediated by FGF23 lead to acceleration of ADPKD progression. Findings obtained in vivo with PCK rats showed deposits of calcium-phosphate crystals induced by a high-phosphate diet in tubule lumens along the corticomedullary junction, which led to increased cytogenesis and disease progression [48]. The present retrospective analysis of urinary phosphate excretion in ADPKD patients treated with dapagliflozin showed that greater changes in urinary phosphate levels were significantly correlated with disease progression, as reflected by the change in htTKV during dapagliflozin treatment. In addition, the findings suggest that monitoring urinary phosphate levels is important during dapagliflozin treatment for patients with ADPKD.

The present study has some limitations. First, the number of patients examined was relatively few, mainly due to the fact that all of the enrolled subjects were treated at a single institution and also because of the low prevalence of ADPKD patients. Second, this was a retrospective observational study, which possibly limits the ability to draw conclusions from the findings. Due to the limited number of participants and retrospective observational design, our findings only demonstrated an association, not direct causality, between dapagliflozin and the disease progression of ADPKD. To confirm the possibility of the effects of dapagliflozin on that progression, additional interventional studies are required. In addition, the observational period was short. Supplemental Table S1 shows eGFR changes after dapagliflozin initiation for relatively long terms. In patients with long-term administration, the decline in eGFR was mild. However, in 8 (40%), dapagliflozin administration was discontinued within six months of initiation due to a decline in eGFR and an increase in TKV. Moreover, TKV change was not evaluated for the entire cohort. Therefore, it is difficult to draw a definitive conclusion based on the present findings regarding the long-term effects of dapagliflozin on kidney function and TKV in ADPKD patients. A large-scale study with a long-term follow-up period would be needed to determine whether the association of dapagliflozin treatment with the progression of ADPKD continues for a long period of time. Third, the method used for measuring TKV in the present study was simple, though a more precise quantitative assessment technique has recently become available. The present measurements of TKV during dapagliflozin administration showed a close similarity to those obtained using the SYNAPSE VINCENT semi-automatic method, recognized to provide accurate results [49] (r = 0.963, *p* < 0.001) (Supplemental Figure S4). Unfortunately, CT images obtained during pre-dapagliflozin treatment could not be analyzed with a newer, more advanced system, as that was not available at our institution. Fourth, this was a single-arm case series study. The absence of a control group cannot distinguish between the effect of the treatment and the natural evaluation of the disease. The reason for the single-arm design of the study was that it was not possible to obtain an appropriate control group. Clinically, repeated CT examinations during a short period are conducted exclusively for ADPKD patients administered with novel treatments including SGLT2 inhibitors. Therefore, short-term TKV changes cannot be evaluated in ADPKD patients without novel treatment. Indeed, a single-arm design has been accepted for recent reports in the field of ADPKD [50,51]. However, it is important to compare our findings with the natural course of ADPKD. A study of disease progression of Japanese ADPKD patients using the Japanese Polycystic Kidney Disease registry (J-PKD) indicated an annual TKV increase of 4.78% and eGFR decline of 5.0% [3], with age, eGFR, and htTKV of the cases in the J-PKD 49 years, 56.7 mL/min/1.73 m^2^, and 825 mL/m, respectively, similar to those for the present patients. In addition, interim results obtained in post-marketing surveillance of tolvaptan in real-world clinical settings, namely SLOW-PKD surveillance, showed an annual TKV increase of 11.7% in rapidly progressive Japanese ADPKD patients whose age, eGFR, and htTKV were 49.7 years, 44.4 mL/min/1.73 m^2^, and 1301 mL/m, respectively [52]. Differences between the present results and the two prior studies of the natural course of ADPKD patients in Japan may support the notion of an accelerated TKV increase during administration of dapagliflozin over a short-term period. Fifth, the effects of SGLT2 inhibitors other than dapagliflozin were not evaluated. A previous study noted that among SGLT2 inhibitors, including ipragliflozin, dapagliflozin, tofogliflozin, canagliflozin, empagliflozin, and luseogliflozin, patients treated with dapagliflozin and ipragliflozin exhibited increased urinary glucose excretion up to 18 h after administration, while that period in patients treated with the others was approximately 12 h [53]. While dapagliflozin is selective for SGLT2, canagliflozin may also have a capacity for SGLT1 inhibition [54]. In consideration of the various pharmacokinetic effects as well as the effect on glucose metabolism of renal cysts of SGLT2 inhibitors, disease progression in ADPKD patients may be more apparent in those treated with dapagliflozin as compared to the others. Sixth, CKD and mineral and bone disorder (CKD-MBD)-related markers were not evaluated in the present study, though they have been reported to be changed after administration of dapagliflozin in both healthy volunteers [55] and type 2 diabetes patients [56]. Serum phosphate, FGF-23, and parathyroid hormone (PTH) levels have been shown to significantly increase, and 1,25-dihydroxyvitamin D levels have decreased with dapagliflozin treatment in type 2 diabetes patients with CKD stages ranging from 2 to 4 [56]. However, those CKD-MBD-related marker levels could not be measured because serum samples were not stored and thus not available. Seventh, the effects of dapagliflozin on heart involvement, including improvement of diastolic dysfunction, could not be investigated since heart ultrasound examinations were not performed during the observation period. SGLT2 inhibitors have been reported to suppress CVD events in CKD patients and improve diastolic dysfunction in patients with type 2 diabetes and those with heart failure [57]. Clinical studies that investigate the effects of SGLT2 inhibitors on heart involvement in ADPKD patients are needed. Eighth, the side effects of SGLT2 inhibitor administration, including infections, could not be appropriately evaluated during the observation period. Although no infection events were noted, a previously published systematic review found that administration of dapagliflozin at 10 mg/day was associated with a significantly increased risk of urinary tract and genital tract infections [58]. Since an ascending urinary tract infection with effects on kidney cysts may have potentially severe consequences for ADPKD patients [59], the detrimental consequences of possible complications, including infections associated with SGLT2 inhibitors given to ADPKD patients, should be carefully evaluated in future studies. Finally, some data, including urinary osmolality, urinary phosphate, urine output, and body weight, were lacking for some of the participants, while changes in food intake such as phosphate load were not evaluated.

## 5. Conclusions

In conclusion, short-term dapagliflozin treatment may be associated with a decrease in eGFR and an increase in htTKV in ADPKD patients. SGLT2 inhibitors may have pleiotropic effects and are considered to be beneficial for patients with ADPKD. To confirm the possibility of the effects of dapagliflozin on ADPKD, additional interventional studies are required.

## Figures and Tables

**Table 1:** Clinical characteristics of study participants.

	No.	
Demographics		
Male/female	20	9/11
Age (years)	20	51 (46–57)
Body mass index (kg/m^2^)	20	23.0 (20.9–24.8)
Systolic blood pressure (mmHg)	20	135 (129–142)
Diastolic blood pressure (mmHg)	19	86 (79–94)
Total kidney volume (mL)	20	967 (689–1168)
Height-adjusted total kidney volume (mL/m)	20	599 (423–707)
Laboratory data		
Blood urea nitrogen (mg/dL)	20	19 (14–22)
Creatinine (mg/dL)	20	1.13 (0.93–1.34)
eGFR (mL/min/1.73 m^2^)	20	47.9 (39.7–56.9)
Hemoglobin (g/dL)	20	13.1 (12.2–14.4)
Serum albumin (g/dL)	20	4.4 (4.2–4.5)
Calcium (mg/dL)	19	9.4 (9.2–9.6)
Phosphate (mg/dL)	19	3.5 (3.3–3.7)
HbA1c (%)	14	5.6 (5.4–5.7)
Plasma glucose (mg/dL)	15	99 (91–133)
Urinary protein (g/gCr)	19	0.08 (0.04–0.17)
Urinary phosphate (g/gCr)	17	0.42 (0.30–0.61)
Complications		
Liver cysts, no. (%)	20	20 (100)
Hypertension, no. (%)	20	14 (70)
Intracranial aneurysms, no. (%)	20	4 (20)
Diabetes, no. (%)	20	1 (5.0)
Medications		
Tolvaptan, no. (%)	20	11 (55)
RAAS inhibitor, no. (%)	20	11 (55)
Calcium channel blocker, no (%)	20	12 (60)
Beta blocker, no (%)	20	4 (20)
Anti-diabetic agent, no (%)	20	1 (5.0)
Phosphorus binder, no (%)	20	1 (5.0)

Values show the number and percentage for categorical variables and the mean ± SD for continuous variables. Abbreviations: eGFR, estimated glomerular filtration rate; HbA1c, hemoglobin A1c: No, number of patients examined; SD, standard deviation; RAAS, renin-angiotensin-aldosterone system.

**Table 2:** Changes in characteristics of study participants after treatment with dapagliflozin.

	No.	Pre	Post	*p* Value
Systolic blood pressure (mmHg)	17	135 (129–143)	127 (121–137)	0.192
Diastolic blood pressure (mmHg)	15	82 (76–90)	85 (80–87)	1.000
Total kidney volume (mL)	20	967 (689–1168)	992 (766–1413)	0.002
Body weight (kg)	11	60 (56–69)	58 (56–69)	0.059
Height-adjusted total kidney volume (mL/m)	20	599 (423–707)	617 (446–827)	0.002
Blood urea nitrogen (g/dL)	20	19 (14–22)	19 (13–21)	0.733
Creatinine (mg/dL)	20	1.13 (0.93–1.34)	1.37 (1.05–1.55)	<0.001
eGFR (mL/min/1.73 m^2^)	20	47.9 (39.7–56.9)	40.8 (33.7–44.5)	<0.001
Hemoglobin (g/dL)	20	13.1 (12.2–14.4)	14.0 (13.1–15.1)	<0.001
Serum albumin (g/dL)	20	4.4 (4.2–4.5)	4.3 (4.2–4.5)	0.818
Calcium (mg/dL)	19	9.4 (9.2–9.6)	9.4 (9.2–9.5)	0.732
Phosphate (mg/dL)	19	3.5 (3.3–3.7)	3.7 (3.3–4.1)	0.068
Urinary protein (g/gCr)	19	0.08 (0.04–0.17)	0.06 (0.00–0.24)	0.118
Urinary phosphate (g/gCr)	16	0.39 (0.29–0.56)	0.45 (0.34–0.56)	0.722
Urinary osmolality (mOsm/kg)	8	329 (126–526)	266 (124–695)	0.813

Values show the number and percentage for categorical variables and mean ± SD for continuous variables. Abbreviations: eGFR, estimated glomerular filtration rate; No., number of patients examined; SD, standard deviation.

**Table 3:** Changes in height-adjusted total kidney volume (htTKV) and estimated glomerular filtration rate (eGFR) of individual study participants after treatment with dapagliflozin.

					Pre-Dapagliflozin		Post-Dapagliflozin	
Pt. No.	Gender	Age	BMI	Tolvaptan	eGFR	htTKV	eGFR	htTKV
1	F	49	27.1	+	24.7	441	23.8	497
2	F	57	23.4	+	32.8	600	27.6	616
3	F	43	18.4	+	34.8	411	30.6	444
4	F	30	26.7	−	35.2	676	25.2	908
5	F	54	18.4	+	38.4	1047	32.1	1101
6	M	45	23.7	−	43.7	680	39.7	715
7	F	52	26.7	−	44.7	355	38.5	363
8	M	52	22.8	−	45.3	438	39.6	445
9	M	48	21.1	−	46.7	419	41.0	447
10	F	60	21.7	−	47.4	665	40.7	672
11	M	48	23.8	+	48.3	732	41.9	892
12	F	62	20.9	+	48.6	243	44.8	268
13	M	42	28.2	+	50.0	648	40.2	743
14	M	62	25.0	−	54.0	175	43.2	168
15	F	52	23.1	+	56.6	592	43.3	554
16	M	57	22.9	+	57.0	1059	43.6	1104
17	M	49	19.9	+	60.6	434	60.2	479
18	M	64	19.6	+	63.7	750	58.8	830
19	F	36	24.3	−	67.3	717	56.2	819
20	F	47	21.6	−	70.2	598	57.9	619

Abbreviations: BMI, body mass index; eGFR, estimated glomerular filtration rate; htTKV, height-adjusted total kidney volume; NA: not available.

**Table 4:** Correlation of change in height-adjusted total kidney volume (htTKV) with clinical factors in ADPKD patients treated with dapagliflozin.

Clinical Variable	No.	r_s_	*p* Value
Blood urea nitrogen	20	−0.076	0.750
eGFR	20	−0.038	0.873
Hemoglobin	20	−0.297	0.204
Serum albumin	20	0.069	0.771
Calcium	19	−0.230	0.343
Phosphate	19	−0.306	0.203
Urinary protein	19	−0.034	0.889
**Urinary phosphate**	**16**	**0.575**	**0.020**

Data include Spearman’s correlation coefficient (r_s_-value) and level of significance (*p* value) (bold *p* < 0.05). Abbreviations: eGFR, estimated glomerular filtration rate; htTKV, height-adjusted total kidney volume; No, number of patients examined.

## Data Availability

Data obtained for this study are available from the corresponding author upon reasonable request.

## Supplementary Materials

The following supporting information can be downloaded at: https://www.mdpi.com/article/10.3390/jcm12196341/s1, Figure S1. Abdominal computed tomography images of representative case; Figure S2. Changes in annual height-adjusted total kidney volume (htTKV) rate in autosomal dominant polycystic kidney disease (ADPKD) patients treated with dapagliflozin; Figure S3. Correlation between change in height-adjusted total kidney volume (htTKV) during dapagliflozin treatment and urinary phosphate in autosomal dominant polycystic kidney disease (ADPKD) patients during dapagliflozin treatment; Figure S4. Correlations between total kidney volume (TKV) calculated using ellipsoid formula and SYNAPSE VINCENT in autosomal dominant polycystic kidney disease (ADPKD) patients during dapagliflozin treatment; Table S1. Long-term changes in estimated glomerular filtration rate (eGFR) in autosomal dominant polycystic kidney disease (ADPKD) patients from pre- to post-dapagliflozin treatment.
