# Supplementary material for: Short-Term Dapagliflozin Administration in Autosomal Dominant Polycystic Kidney Disease—A Retrospective Single-Arm Case Series Study
Source: J Clin Med. 2023 Oct 3;12(19):6341. doi: 10.3390/jcm12196341 (PMC10573882; doi:10.3390/jcm12196341)
Supplement: Supplementary file 1 [file jcm-12-06341-s001.zip › jcm-2621575-supplementary.pdf]

Journal of
Clinical Medicine

MDPI

Supplementary Material

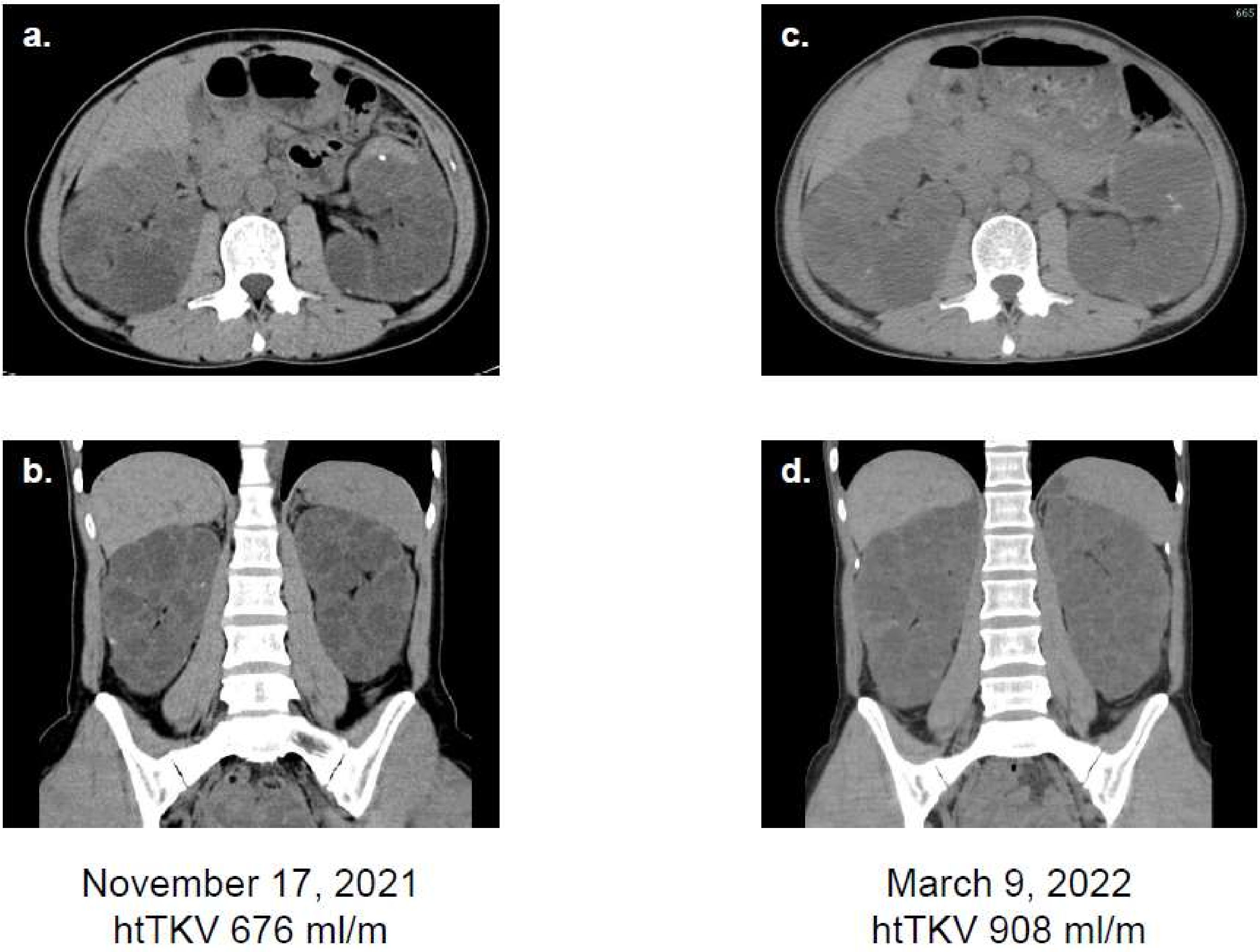

Figure S1. Abdominal computed tomography images of representative case. a, b. Dapagliflozin administration was started on November 17, 2021 and height-adjusted total kidney volume (htTKV) was 676 ml/m. c, d. That volume continued to increase during the dapagliflozin treatment period to 908 ml/m on March 9, 2022.

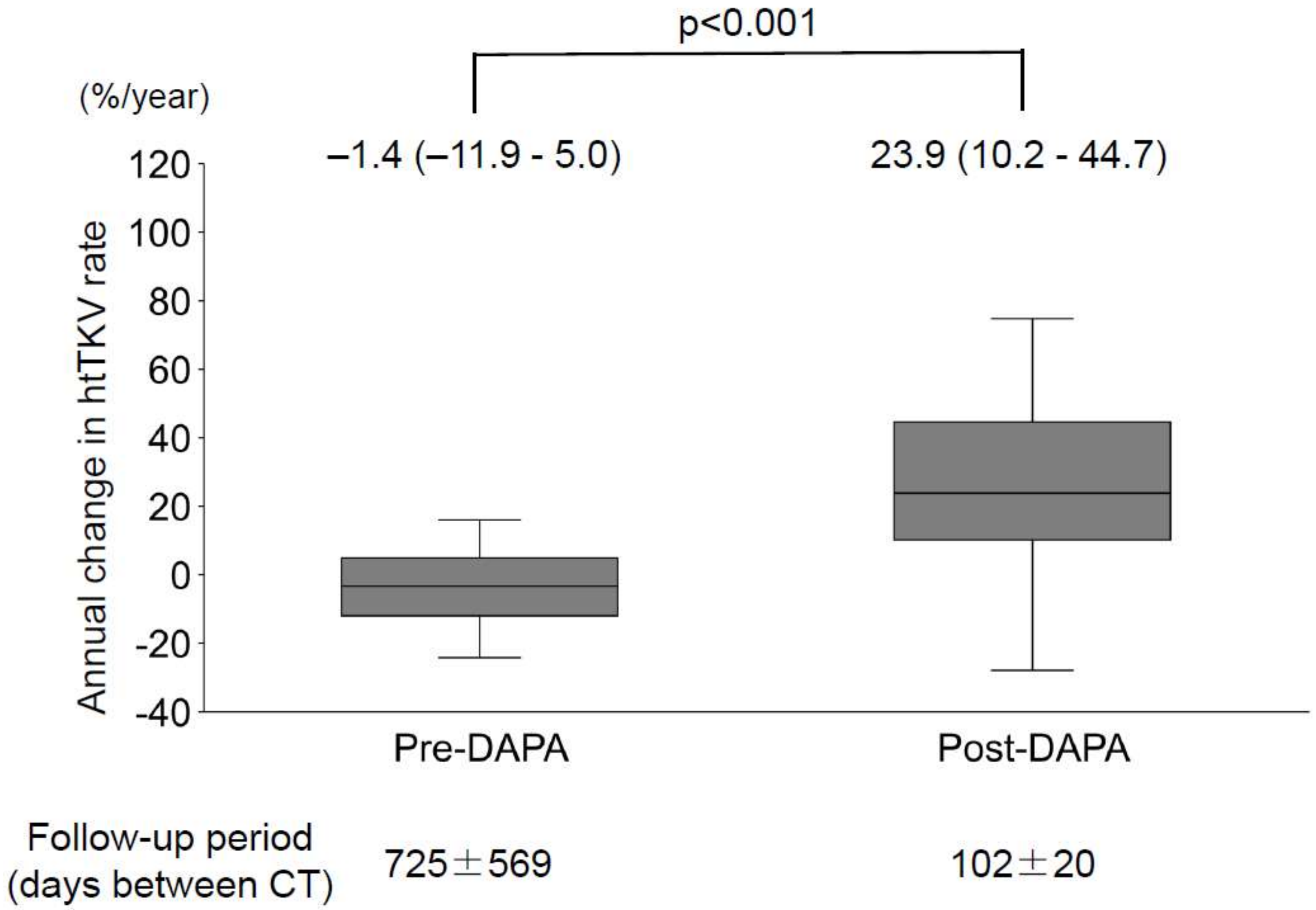

Figure S2. Changes in annual height-adjusted total kidney volume (htTKV) rate in autosomal dominant polycystic kidney disease (ADPKD) patients treated with dapagliflozin. Dapagliflozin treatment significantly accelerated kidney enlargement in the ADPKD patients. The boxes indicate minimum, the 25th, 50th, 75th percentile levels, and whiskers indicate minimum and maximum. pre-DAPA: pre-dapagliflozin treatment, post-DAPA: post dapagliflozin treatment

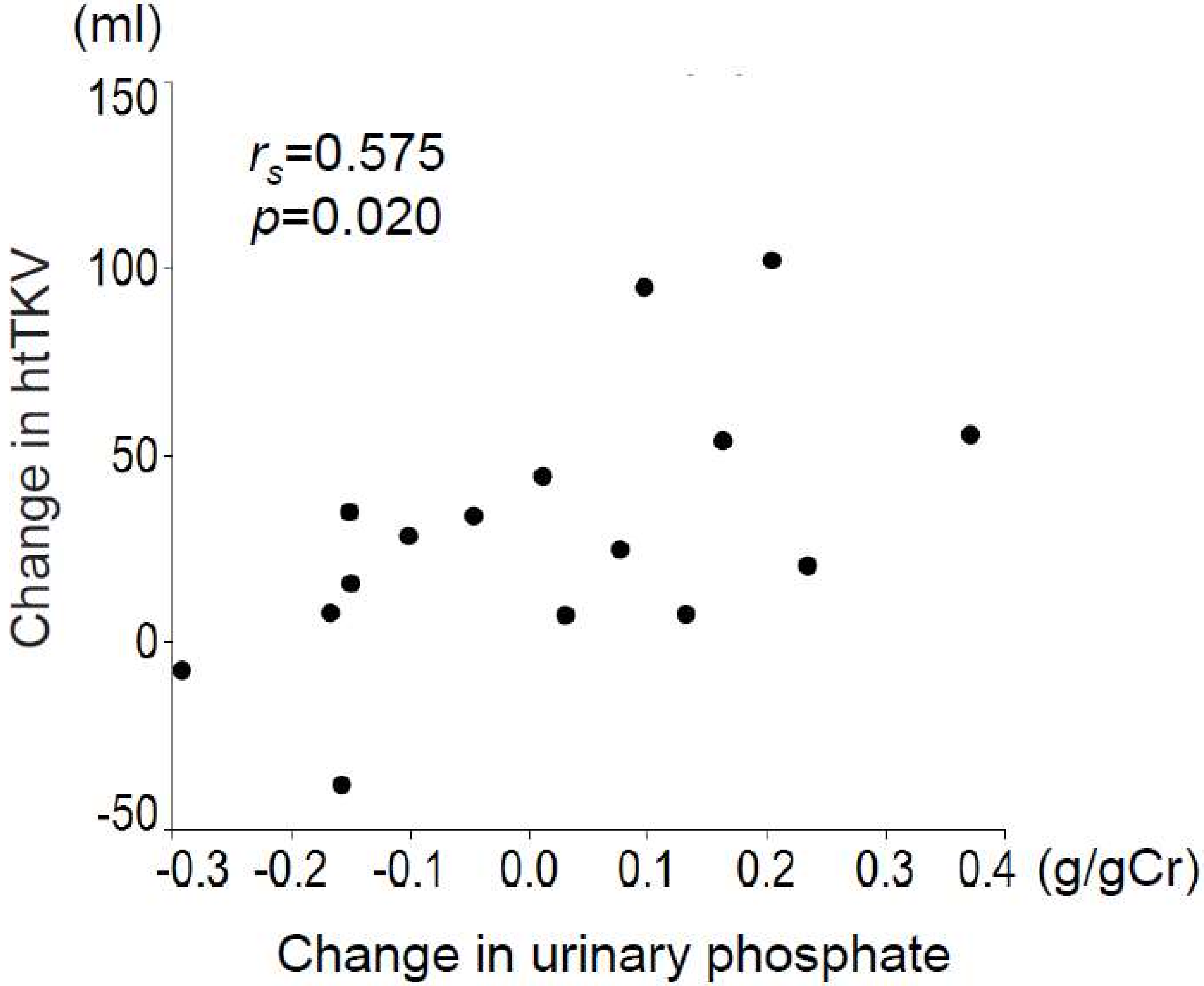

Figure S3. Correlation between change in height-adjusted total kidney volume (htTKV) during dapagliflozin treatment and urinary phosphate in autosomal dominant polycystic kidney disease (ADPKD) patients during dapagliflozin treatment. Change in urinary phosphate level showed a significant correlation with change in htTKV. Abbreviations: r_s, Spearman's correlation coefficient; P, level of significance.

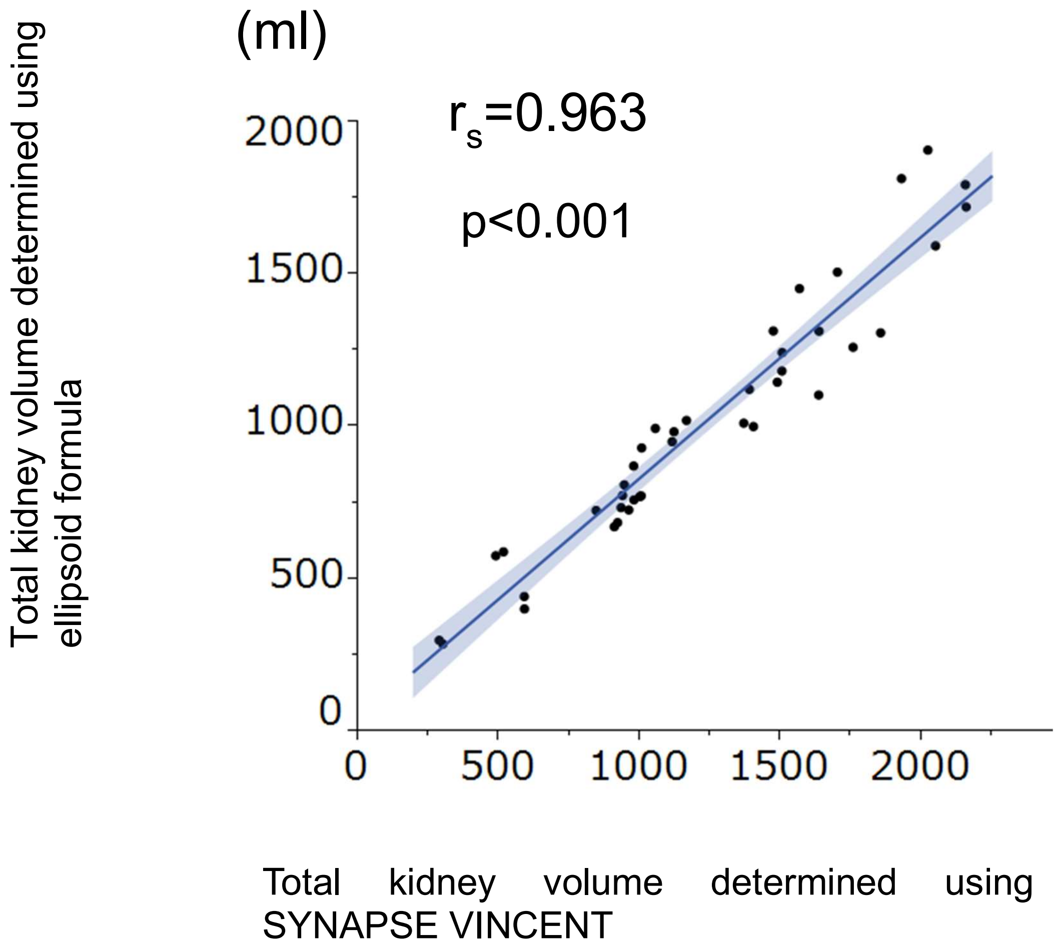

Figure S4. Correlations between total kidney volume (TKV) calculated using ellipsoid formula and SYNAPSE VINCENT in autosomal dominant polycystic kidney disease (ADPKD) patients during dapagliflozin treatment. Abbreviations: r_s, Spearman's correlation coefficient; P, level of significance

Table S1. Long-term changes in estimated glomerular filtration rate (eGFR) in autosomal dominant polycystic kidney disease (ADPKD) patients from pre- to post-dapagliflozin treatment.

Pt. no.	Pre-dapagliflozin eGFR mL/min/1.73 m^2	Post-dapagliflozin eGFR mL/min/1.73 m^2 (days after dapagliflozin administration)		Discontinuation/continuation of dapagliflozin
	First CT	First visit	Last visit	
1	24.7	23.9 (33)	21.6 (222)	Discontinuation
2	32.8	27.6 (82)	27.8 (161)	Discontinuation
3	34.8	32.2 (42)	28.0 (534)	Discontinuation
4	35.2	25.2 (112)	22.2 (205)	Discontinuation
5	38.4	34.0 (63)	31.6 (180)	Discontinuation
6	43.7	39.7 (84)	37.6 (476)	Continuation
7	44.7	41.6 (28)	38.5 (126)	Discontinuation
8	45.3	38.4 (62)	38.0 (524)	Discontinuation
9	46.7	41.0 (84)	37.9 (161)	Discontinuation
10	47.4	46.3 (42)	42.2(357)	Continuation
11	48.3	44.2 (28)	41.9 (107)	Discontinuation
12	48.6	47.5 (44)	39.3 (464)	Discontinuation
13	50.0	42.0 (59)	41.7 (587)	Discontinuation
14	54.0	51.9 (42)	44.1 (541)	Continuation
15	56.6	46.1 (28)	43.3 (84)	Discontinuation
16	57.0	49.4 (35)	43.0 (562)	Discontinuation
17	60.6	60.2 (84)	57.8 (175)	Discontinuation
18	63.7	58.8 (61)	49.8 (234)	Discontinuation
19	67.3	56.2 (84)	51.4 (340)	Discontinuation
20	70.2	65.6 (32)	56.4 (287)	Discontinuation
